# Clinical and Echocardiographic Insights Into Right Heart Masses: A Retrospective Study in China (2010–2023)

**DOI:** 10.1002/cam4.71530

**Published:** 2026-01-15

**Authors:** Lei Liu, Peng Teng, Fudong Fan, Hui Chen, Yanfei Mo, Jing Yao, Xuelin Yang, Aijuan Fang

**Affiliations:** ^1^ Department of Ultrasound Medicine Drum Tower Hospital Affiliated to Nanjing University Medical School Nanjing China; ^2^ Chinese Academy of Medical Sciences and Peking Union Medical College Beijing China; ^3^ Department of Cardiac Surgery Affiliated First Hospital of Zhejiang University Medical School Hangzhou China; ^4^ Department of Cardiac Surgery Drum Tower Hospital Affiliated to Nanjing University Medical School Nanjing China; ^5^ Department of Cardiology Pukou Hospital of Chinese Medicine Affiliated to China Pharmaceutical University Nanjing China; ^6^ Department of Anesthesiology Drum Tower Hospital Affiliated to Nanjing University Medical School Nanjing China

**Keywords:** cardiac tumors, echocardiography, pathology, prognosis, right heart, survival analysis

## Abstract

**Background:**

This study aimed to analyze the clinical and echocardiographic characteristics, pathological profiles, and outcomes of right heart masses, and to explore the role of echocardiography in the evaluation of these masses.

**Methods:**

We retrospectively analyzed 171 patients with echocardiographically diagnosed and pathologically confirmed right heart masses from two centers (2010–2023). Data on clinical presentation, echocardiographic features, pathology, and follow‐up were collected. Survival analysis using Kaplan–Meier curves and multivariable Cox regression was performed to identify prognostic factors.

**Results:**

Among 171 patients, 114 had primary cardiac tumors (82 benign, 30 malignant, and 2 of uncertain biological behavior), 41 had metastatic tumors, and 16 had thrombi or other lesions. Myxomas (63.4%) and angiosarcomas (63.3%) were the most common benign and malignant tumors, respectively. Patients with benign tumors had significantly better survival than those with malignant tumors, metastases, or thrombi (all *p* < 0.01). In the primary tumor subgroup (*n* = 112), better survival was associated with pedunculated morphology, well‐defined margins, high mobility, and absence of metastasis (all *p* < 0.01). Multivariable analysis identified malignant tumor type (HR = 10.072, *p* < 0.001; reference: benign tumor), absence of a pedicle (HR = 2.610, *p* = 0.044; reference: presence of a pedicle), and presence of metastasis (HR = 3.210, *p* = 0.025; reference: no metastasis) as independent prognostic factors. The median follow‐up was 26 months, with a recurrence rate of 4.7% and overall mortality of 35.7%.

**Conclusion:**

Tumor type, pedicle presence, and metastasis are key prognostic factors for primary right heart tumors. Echocardiography is essential for diagnosis and differential diagnosis of right heart masses.

## 1 Introduction

Right heart masses encompass a spectrum of rare but clinically significant entities in cardiology, including benign and malignant tumors, thrombi, and other pathological formations [1]. These lesions may arise from primary cardiac tumors, metastatic deposits, or nonneoplastic conditions such as thrombi and cysts, each presenting distinct diagnostic and therapeutic challenges [2]. While primary cardiac tumors exhibit an exceptionally low incidence (0.001%–0.03% in autopsy series), their clinical relevance stems from potential complications including hemodynamic obstruction, embolic events, arrhythmias, and cardiac failure [3]. Myxomas dominate the benign category, whereas sarcomas account for the majority of malignant cases [2]. Nonneoplastic masses, particularly organized thrombi, pose diagnostic dilemmas due to overlapping imaging features with true neoplasms [4]. Compared to left heart masses, right‐sided lesions demonstrate a higher prevalence of malignancies, more aggressive biological behavior, and frequent metastatic involvement [2]. Clinical manifestations range from incidental detection to severe presentations like dyspnea, chest pain, or syncope, underscoring the imperative for early diagnosis to guide management strategies such as surgical resection, biopsy, or anticoagulation [5]. Prognosis varies substantially by etiology, with malignant tumors typically conferring poor outcomes despite intensive therapies [2]. Given the diagnostic complexity and rarity of these masses, systematic analyses of their clinical profiles, echocardiographic characteristics, histopathological correlations, and longitudinal outcomes are critical. Such investigations enhance our understanding of disease mechanisms while clarifying the diagnostic utility and constraints of echocardiography [1, 4, 5, 6].

Echocardiography, particularly transthoracic (TTE) and transesophageal (TEE) modalities, serves as the cornerstone imaging technique for evaluating right heart masses [1]. This technology enables real‐time structural assessment, including mass localization, dimensions, mobility, attachment characteristics, and hemodynamic consequences [5]. Recent advancements in three‐dimensional echocardiography and contrast‐enhanced imaging have further refined diagnostic capabilities [6]. Nevertheless, differentiating benign from malignant lesions or distinguishing tumors from thrombi persists as a formidable challenge, necessitating multidisciplinary integration of clinical, imaging, and histopathological data [6]. However, differentiating benign from malignant lesions or distinguishing tumors from thrombi remains a significant challenge, necessitating a comprehensive, multidisciplinary approach that integrates clinical, imaging, and pathological findings [6]. This study aims to provide a comprehensive analysis of a large patient cohort with right heart masses, focusing on clinical‐echocardiographic correlations, histopathological diagnoses, and long‐term outcomes. The study also prioritizes the identification of key echocardiographic features that aid in differential diagnosis and prognosis.

## 2 Materials and Methods

### 2.1 Study Population

**FIGURE 1:**
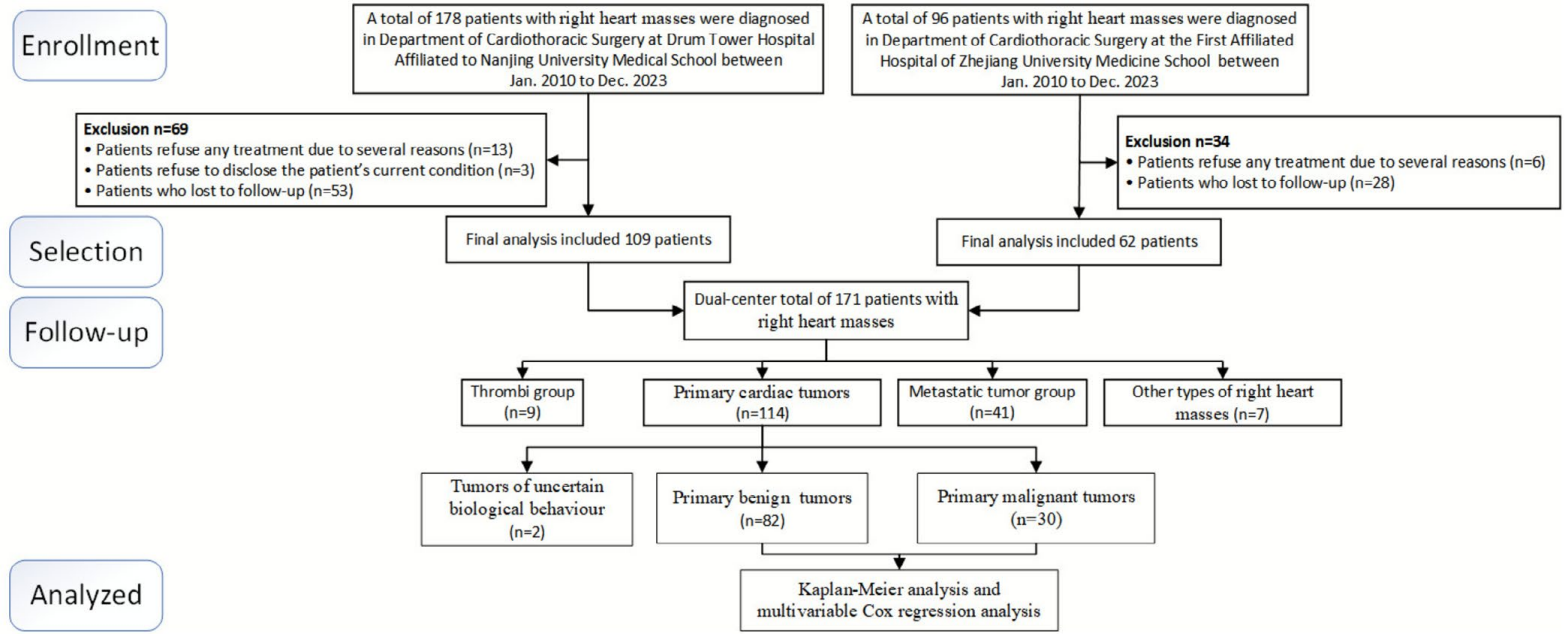
Flow diagram of the case selection procedure.

This retrospective study included 171 patients diagnosed with right heart masses via TTE between January 2010 and December 2023 at Nanjing Drum Tower Hospital and the First Affiliated Hospital of Zhejiang University (Figure 1). The cohort consisted of 89 males (52.0%) and 82 females (48.0%), aged 14–87 years (mean age: 57.1 ± 14.2 years). All patients underwent TTE upon admission, followed by surgical resection or biopsy for pathological confirmation. It must be noted that this inclusion criterion may introduce selection bias, as the present cohort primarily represents lesions that underwent surgical intervention or biopsy and may not fully represent the entire spectrum of right heart masses (e.g., typical thrombi diagnosed based on clinical and imaging follow‐up). Clinical data, including medical history, symptoms, signs, electrocardiograms, blood tests, and NT‐proBNP levels, were collected. Additional imaging studies, such as enhanced chest CT and contrast echocardiography, were performed when necessary to further characterize the masses.

### 2.2 Study Design

This study was designed as a dual‐center, retrospective cohort study to evaluate the clinical, echocardiographic, and pathological characteristics of right heart masses, as well as their prognostic implications. The study was divided into three phases: (1) initial clinical and echocardiographic characterization, (2) pathological confirmation and classification of masses, and (3) follow‐up for recurrence, survival, and treatment outcomes.

TTE was performed using Philips EPIQ 7C, Philips CVx, and GE E95 color Doppler ultrasound systems with probe frequencies of 1.0–5.0 MHz. Patients were examined in the left lateral decubitus position, and multi‐plane imaging was used to evaluate the location, size, echogenicity, morphology, attachment, mobility, and relationship of the masses to surrounding structures. Color and spectral Doppler were used to assess hemodynamic changes, and pericardial effusion was noted. Chamber dimensions and cardiac function were routinely measured. The images for each case were reviewed jointly by at least two experienced cardiologists specializing in echocardiography to reach a consensus interpretation regarding the mass characteristics. For inconclusive cases, contrast‐enhanced ultrasound with SonoVue (Bracco, Italy) was performed to assess vascularity and differentiate between benign and malignant masses. Enhanced chest CT was used to evaluate extracardiac involvement, pulmonary embolism, and distant metastases. Pathological results served as the diagnostic gold standard. Masses were classified into primary benign, primary malignant, metastatic, and nonneoplastic lesions. Echocardiographic features used to characterize a mass as suggestive of malignancy in this study included: (1) Morphology: irregular or infiltrative borders with destruction of normal tissue planes; (2) Attachment: a broad‐based attachment to the cardiac wall without a discrete stalk; (3) Composition: heterogeneous echotexture, often with areas suggesting necrosis or hemorrhage; (4) Pericardial involvement: presence of a pericardial effusion, particularly if hemorrhagic, or direct tumor extension into the pericardium; (5) Dynamic behavior: rapid interval growth on serial examinations (when available); and (6) Multiplicity: presence of multiple, distinct masses within the right heart chambers or associated great vessels. It is critical to note that these criteria are indicative rather than definitive. A final diagnosis of malignancy was always established by histopathological examination. The diagnosis of thrombus was established by the following criteria: the majority of thrombi (7 out of 9) were confirmed by pathological examination after surgical removal. For the remaining two cases not undergoing surgery, the diagnosis was based on a combination of criteria: (1) appearance as an avascular mass on transthoracic and/or transesophageal echocardiography (confirmed by contrast echocardiography if necessary); (2) the presence of clear predisposing factors for thrombosis (e.g., atrial fibrillation, heart failure); and (3) significant reduction in size or complete resolution of the mass on follow‐up echocardiography after initiation of appropriate anticoagulation therapy. Follow‐up data were collected to evaluate recurrence, survival, and treatment outcomes. The follow‐up period ranged from 1 to 120 months, with a median duration of 26 months.

### 2.3 Data Collection

Clinical data included demographic information (age, sex, BMI), presenting symptoms, New York Heart Association (NYHA) Functional Classification, and comorbidities (e.g., congenital heart disease, uterine fibroids, pericardial effusion and/or pleural effusion, pulmonary embolism, hypertension, diabetes, and hyperlipidemia). Echocardiographic parameters included mass size, location, attachment, mobility, echogenicity, and hemodynamic effects (e.g., tricuspid valve obstruction, regurgitation). Pathological data included histological classification and evidence of metastasis. Surgical and follow‐up data included type of surgery, recurrence, and survival outcomes.

### 2.4 Statistical Analysis

Statistical analyses were performed using SPSS 22.0, and GraphPad Prism 9.5 was used for visualization. Continuous variables with normal distribution were expressed as mean ± standard deviation ($\bar{x}$ ± s) and compared using independent sample *t*‐tests. Non‐normally distributed data were expressed as median (interquartile range, M[QR]) and analyzed using the Mann–Whitney *U* test. Categorical variables were expressed as frequencies and percentages and compared using the chi‐square or Fisher's exact test. Kaplan–Meier analysis was used to assess survival rates, while multivariable Cox regression analysis identified independent prognostic factors. A *p*‐value < 0.05 was considered statistically significant.

For the multivariable Cox proportional hazards regression analysis, all potential clinical and echocardiographic prognostic variables were first assessed in univariable analyses. Variables with a *p*‐value < 0.1 in the univariable analysis were considered for inclusion in the initial multivariable model. Given their established role as important prognostic confounders, age and sex were forced into the model regardless of their univariable significance. The final model was built using the Enter method. Prior to modeling, the proportional hazards assumption was verified for all candidate variables by examining the correlation between Schoenfeld residuals and time; the global test was nonsignificant (*p* > 0.05), indicating that the assumption was met. Multicollinearity among the variables included in the final model was assessed using the variance inflation factor (VIF); all VIF values were below 2, suggesting that multicollinearity was not a concern. It is important to note that the primary survival analyses, including the multivariable Cox regression, were performed on the subgroup of 114 patients with primary cardiac tumors. This focus was chosen because the pathophysiology and key prognostic determinants of metastatic tumors and thrombi are fundamentally different from those of primary tumors. Survival analysis results for the entire cohort (*n* = 171) are available from the corresponding author upon reasonable request.

### 2.5 Ethical Approval and Informed Consent

This study protocol was approved by the Medical Ethics Committee of Nanjing University Medical School Affiliated Drum Tower Hospital (Approval No. 2022‐210‐01). In accordance with institutional guidelines for retrospective analyses of anonymized clinical data, the committee granted a waiver of the requirement for written informed consent.

## 3 Results

### 3.1 Clinical Presentation

The study included 171 patients with an average age of 57.1 ± 14.2 years, ranging from 14 to 87 years. Among them, 48 (28.1%) were asymptomatic, with right atrial masses detected during routine physical examinations. The most common initial symptom was chest tightness (52 cases, 30.4%), followed by palpitations (21 cases, 12.3%), chest pain (19 cases, 11.1%), lower limb edema (17 cases, 9.9%), cough (11 cases, 6.4%), and syncope (three cases, 1.8%). The average heart rate was 83.4 ± 16.1 beats per minute. Abnormal heart rhythms included sinus tachycardia (32 cases), sinus bradycardia (18 cases), atrial fibrillation (seven cases), atrial flutter (three cases), frequent atrial premature contractions (three cases), and frequent ventricular premature contractions (five cases). Enhanced chest CT revealed secondary pulmonary embolism in nine patients, of which six were confirmed as myxomas (likely tumor emboli) and three as thrombi with partial detachment (Table 1).

**TABLE 1:** Demographic and clinical characteristics.

Indicator	Frequency, *n* (%)
Gender	
Male	89 (52.0%)
Female	82 (48.0%)
Age (mean ± standard deviation, years)	57.1 ± 14.2
NYHA functional classification	
Class I	82 (48.0%)
Class II	42 (24.5%)
Class III	36 (21.1%)
Class IV	11 (6.4%)
Comorbidities	
Congenital heart disease	12 (7.0%)
Uterine fibroids	23 (13.5%)
Pericardial effusion and/or pleural effusion	20 (11.7%)
Pulmonary embolism	31 (18.1%)
Hypertension	39 (22.8%)
Diabetes	31 (18.1%)
Hyperlipidemia	27 (15.8%)
Initial symptoms	
Asymptomatic	48 (28.1%)
Chest tightness	52 (30.4%)
Chest pain	19 (11.1%)
Palpitations	21 (12.3%)
Lower extremity edema	17 (9.9%)
Cough	11 (6.4%)
Dizziness	8 (4.7%)
Syncope	3 (1.8%)
Area of involvement	
Single mass	140 (81.9%)
Multiple mass	31 (18.1%)
Surgical procedures	
First surgery	167 (97.7%)
Second surgery	4 (2.3%)
Pathological biopsy	5 (2.9%)
Follow‐up	
Deaths	61 (35.7%)
Recurrence	12 (4.1%)
Follow‐up time [median (QR), months]	26 (15, 43)

Abbreviation: NYHA, New York Heart Association, USA.

### 3.2 Pathological Results of Right Heart Masses

Among the 171 patients, 114 had primary cardiac tumors, including 82 benign, 30 malignant, and 2 of uncertain biological behavior. Metastatic tumors were identified in 41 patients, of which 38 were malignant and 3 were benign uterine leiomyomas. Nine patients had organized thrombi or highly mobile thrombi, five had proliferative fibrous collagenous tissue, one had proliferative lymphoid tissue, and one had a right atrial blood cyst. Myxomas were the most common benign tumors (52 cases, 63.4%). Malignant tumors included 19 angiosarcomas, six primary cardiac lymphomas, four intimal sarcomas, and one soft tissue sarcoma. Multiple masses were observed in 31 patients, including 13 involving the inferior vena cava and right atrium, 5 involving the right atrium and right ventricle, 4 involving the pericardium and right atrium, 3 involving the pericardium and right ventricle, 3 involving the superior vena cava and right atrium, 1 involving the right atrium, right ventricle, and pulmonary artery, and 2 with bilateral atrial myxomas (Table 2).

**TABLE 2:** Pathological results.

Pathological results	Cases (*n*, %)	Multiple mass (*n*)
Primary benign tumor	82 (49.8%)	—
Myxoma	52 (30.4%)	2
Hemangioma	11 (6.4%)	—
Papillary elastofibroma	9 (5.3%)	—
Lipoma	7 (4.1%)	—
Smooth muscle tumor	3 (1.8%)	3
Primary malignant tumor	30 (17.4%)	—
Angiosarcoma	19 (11.1%)	8
Lymphoma	6 (3.5%)	1
Intimal sarcoma	4 (2.3%)	—
Soft tissue sarcoma	1 (0.6%)	1
Dynamically uncertain tumor of intermediate malignancy	2 (1.2%)	—
Metastatic tumor	38 (22.2%)	14
Thrombus	9 (5.3%)	—
Encapsulated organized thrombus	6 (3.5%)	—
High‐mobility thrombus	3 (1.8%)	2
Other types of lesions	7 (4.1%)	—
Collagenous tissue hyperplasia	5 (2.9%)	—
Lymphoid hyperplasia	1 (0.6%)	—
Right atrial cyst	1 (0.6%)	—

### 3.3 Echocardiographic Features of Right Heart Masses

**FIGURE 2:**
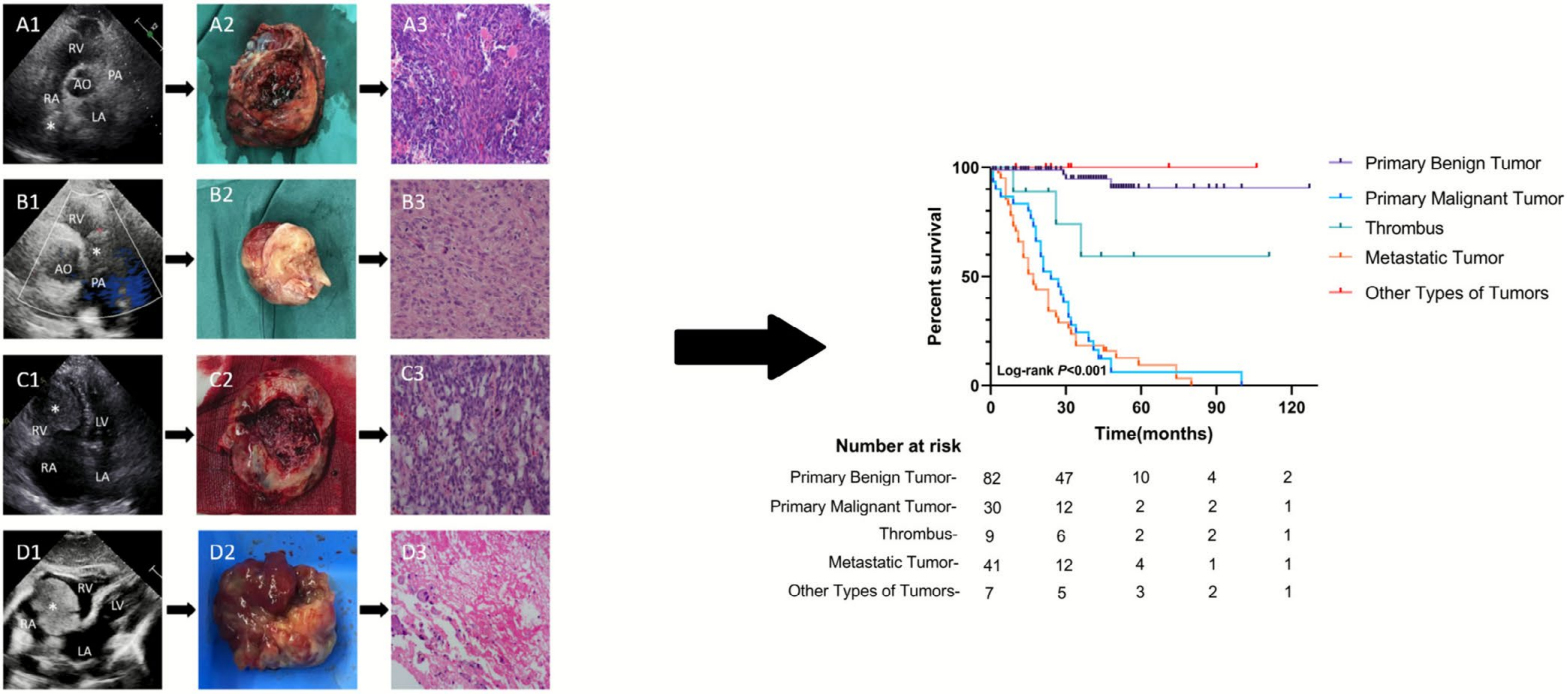
Central illustration. Representative echocardiographic and pathological features of major right heart masses. (A1, A2) Right ventricular angiosarcoma. (B1, B2) Intimal sarcoma of the pulmonary artery. (C1, C2) Clear cell carcinoma of the kidney has spread to the right ventricle of the heart. (D1, D2) Right atrial myxoma. (A3–D3) All pathological pictures are HE staining, 200× times. Ao, aorta; HE, hematoxylin and eosin; LA, left atrium; LV, left ventricle; RA, right atrium; RV, right ventricle.

Among the 171 patients, 38 had right atrial masses causing tricuspid valve obstruction, 13 had inferior vena cava obstruction, and 3 had superior vena cava obstruction. Moderate or severe tricuspid regurgitation was observed in 20 patients, including 11 cases of malignant tumors invading the tricuspid valve, 8 cases of large myxomas impairing tricuspid valve function, and 1 case of a blood cyst located on the anterior leaflet of the tricuspid valve causing valve insufficiency. Three cases of intravenous leiomyomas extended along the inferior vena cava into the right atrium, and 3 myxomas were attached to the lower edge of the interatrial septum. Representative echocardiographic examples of the main mass subtypes are provided in the Central Illustration (Figure 2), highlighting characteristic morphological features.

Contrast echocardiography was performed in 19 patients. Among them, eight lesions showed significant contrast enhancement, suggesting hypervascular masses diagnosed as malignant tumors, with a diagnostic accuracy of 87.5% (seven cases). Nine lesions showed partial contrast enhancement, suggesting hypovascular masses diagnosed as benign tumors, with a diagnostic accuracy of 77.8% (seven cases). Two lesions showed no contrast enhancement, suggesting avascular masses diagnosed as thrombi, consistent with pathological results (two cases, 100%).

Distant metastases were observed in 13 patients. Among these, four angiosarcomas invaded the entire heart wall and pericardium, with metastases to both lungs, bilateral adrenal glands, and mediastinal lymph nodes. Another four angiosarcomas invaded the entire heart wall and pericardium without distant metastases. Three angiosarcomas metastasized to cervical lymph nodes, and two intimal sarcomas metastasized to both lungs.

**FIGURE 3:**
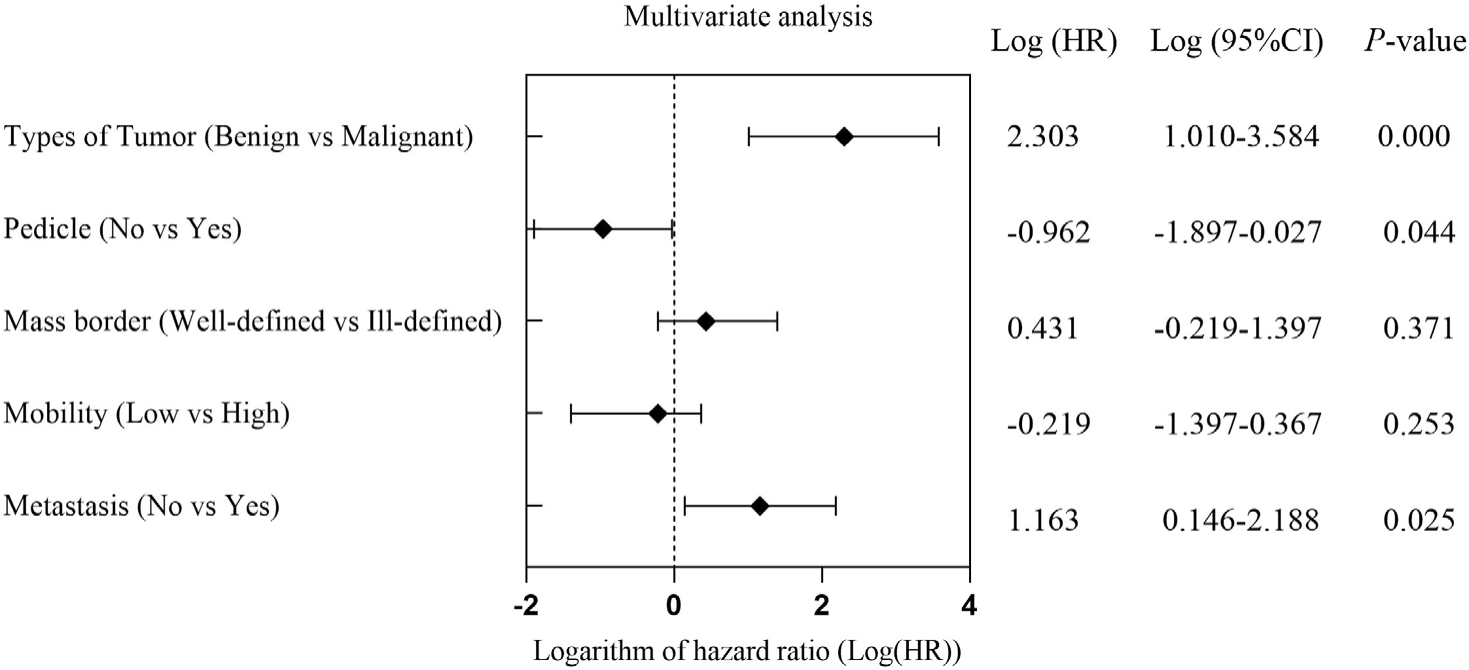
Multivariate analysis for independent prognostic factors.

A comparison of echocardiographic features of 112 of these primary benign and malignant tumors revealed statistically significant differences in tumor type, pedicle presence, margin clarity, mobility, and metastasis (*p* < 0.01). However, no significant differences were observed in age, sex, BMI, tumor location, tricuspid valve obstruction, pericardial effusion, or pulmonary embolism (*p* > 0.05). Multivariable Cox regression analysis identified primary malignant tumors (Hazard Ratio (HR) = 10.072, 95% CI: 2.753, 36.852, *p* < 0.001), absence of a pedicle (HR = 0.383, 95% CI: 0.150, 0.973, *p* = 0.044), and metastases (HR = 3.210, 95% CI: 1.159, 8.892, *p* = 0.025) as independent predictors of mortality in patients with right heart masses (Table 3 and Figure 3).

**TABLE 3:** High risk factors for death from primary tumors of the right heart.

Item	Univariate Cox		Multivariate Cox	
	HR (95% CI)	*p*	HR (95% CI)	*p*
Age				
Young adults and adolescents (< 35)	Reference			
Middle‐aged adults (≥ 35, < 65)	0.425 (0.107–1.698)	0.226	—	
Older adults (≥ 65)	0.349 (0.080–1.527)	0.162	—	
Gender				
Female	Reference			
Male	1.653 (0.812–3.363)	0.166	—	
BMI				
Underweight (< 18.5)	Reference			
Normal weight (≥ 18.5, < 24.9)	0.773 (0.241–2.476)	0.664	—	
Overweight (≥ 25)	0.244 (0.047–1.261)	0.092	—	
Location of the mass				
RA	Reference			
RV	0.439 (0.140–1.384)	0.160	—	
Other locations in the right cardiac system	1.237 (0.395–3.878)	0.715	—	
Mass type				
Primary benign tumor	Reference			
Primary malignant tumor	56.620 (23.680–135.400)	< 0.001	10.072 (2.753–36.852)	< 0.001
Thrombus	41.420 (3.441–498.500)	0.003		
Metastatic tumor	32.770 (16.380–65.590)	< 0.001		
Other types of tumor	0.338 (0.009–13.020)	0.560		
Whether pedicle				
No	Reference			
Yes	0.252 (0.123–0.520)	< 0.001	0.383 (0.150–0.973)	0.044
Mass border definition				
Ill‐defined	Reference			
Well‐defined	0.086 (0.039–0.192)	< 0.001	NS	
Whether pericardial effusion				
No	Reference			
Yes	1.077 (0.501–2.315)	0.849	—	
Whether tricuspid valve obstruction				
No	Reference			
Yes	1.386 (0.598–3.215)	0.447	—	
Whether pulmonary embolism				
No	Reference			
Yes	2.428 (0.838–7.038)	0.102	—	
Mass mobility				
High	Reference			
Low	3.000 (1.458–6.175)	0.003	NS	
Metastasis				
No	Reference			
Yes	81.130 (28.740–229.100)	< 0.001	3.210 (1.159–8.892)	0.025

Abbreviations: BMI, body mass index; CI, confidence interval; HR, hazard ratio; NS, not significant.

### 3.4 Surgical Treatment

A total of 166 patients underwent complete or partial tumor resection under cardiopulmonary bypass or deep hypothermic circulatory arrest. Due to tumor involvement of the tricuspid valve, 11 patients underwent tricuspid valve repair, and 2 underwent tricuspid valve bioprosthetic replacement. Five patients underwent biopsy for pathological examination.

### 3.5 Follow‐Up Results

Follow‐up data were available for 87.2% (171) of the patients, with a median follow‐up duration of 26 (15, 43) months. During follow‐up, 73 patients experienced recurrence or death. Recurrence occurred in 12 cases, including 3 myxomas, 3 metastatic tumors, 3 angiosarcomas, 1 soft tissue sarcoma, 1 lymphoma, and 1 intimal sarcoma. There were 61 deaths, including 36 from metastatic tumors, 16 from angiosarcomas, 3 from intimal sarcomas, and 2 from lymphomas. Causes of death included heart failure, multi‐organ failure, arterial embolism, or malignant arrhythmias. Three thrombus patients died, likely due to heart failure, and 1 hemangioma patient died from age‐related postoperative complications.

### 3.6 Survival Differences Among Various Types of Right Heart Tumors

**FIGURE 4:**
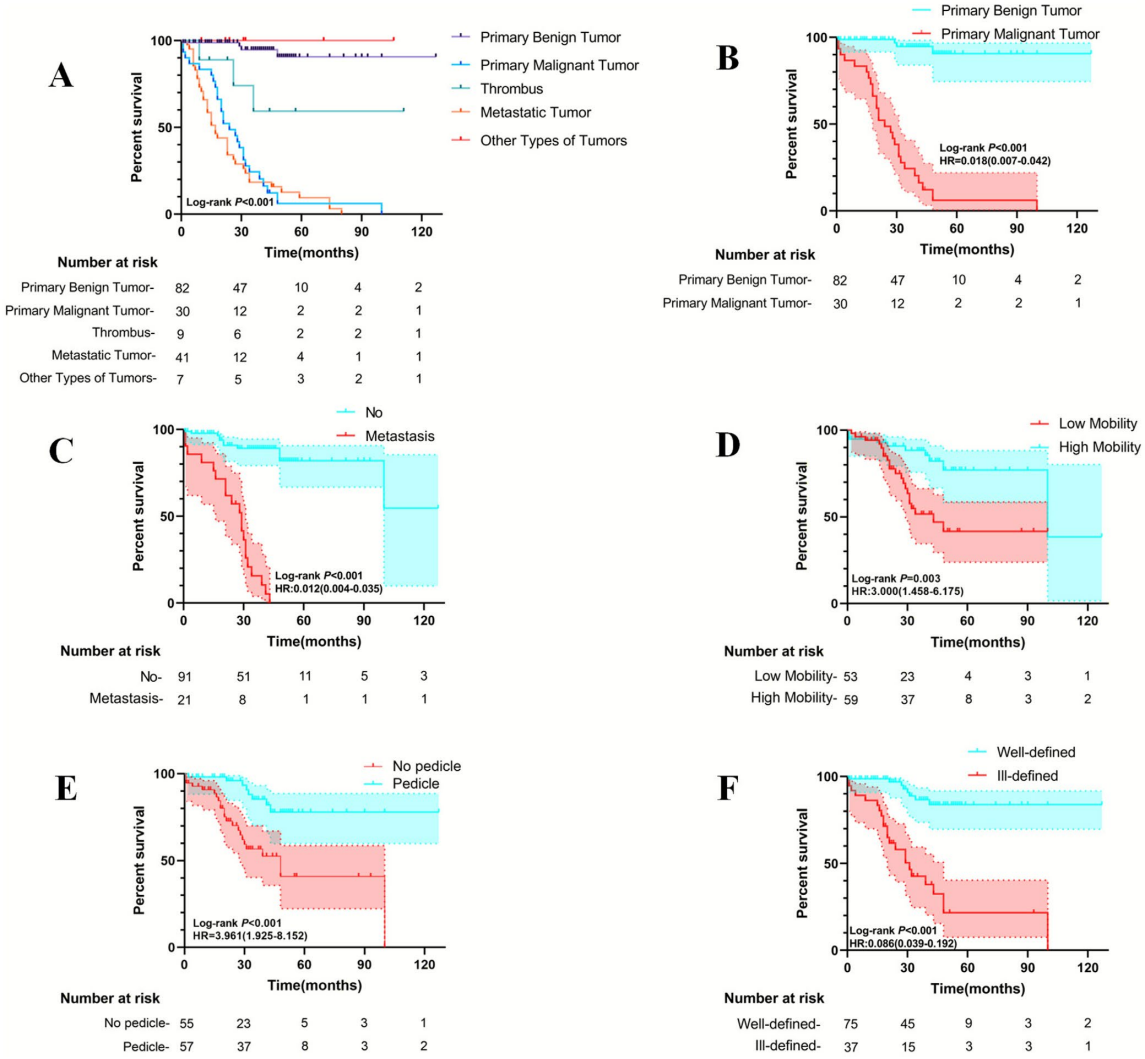
Kaplan–Meier curves of overall survival for prognostic factors.

Significant differences in survival rates were observed among patients with primary benign tumors, primary malignant tumors, thrombi, metastatic tumors, and other tumor types (log‐rank *p* < 0.001). These findings indicate that tumor type substantially influences prognosis in patients with right heart tumors (Figure 4A).

### 3.7 Comparison of Survival Between Primary Benign and Primary Malignant Tumors

Survival rates differed markedly between patients with primary benign and primary malignant tumors (log‐rank *p* < 0.001) (Figure 4B). Cox regression analysis revealed a significantly lower mortality risk in the benign tumor group compared to the malignant tumor group (HR = 0.018, 95% CI: 0.007–0.042), demonstrating a substantially better prognosis for patients with primary benign tumors.

### 3.8 Survival Comparison Between Patients With and Without Tumor Metastasis

A significant difference in prognosis was evident between patients with and without metastasis (log‐rank *p* < 0.001) (Figure 4C). Patients with metastasis had a significantly higher mortality risk compared to those without (HR = 81.130, 95% CI: 28.740–229.100), indicating a clear survival advantage in the absence of metastasis.

### 3.9 Survival Comparison Between High‐ and Low‐Mobility Tumors

Patients with low‐mobility tumors demonstrated significantly lower survival rates than those with high‐mobility tumors (log‐rank *p* = 0.003) (Figure 4D). The mortality risk in the low‐mobility group was threefold higher than in the high‐mobility group (HR = 3.000, 95% CI: 1.458–6.175), suggesting that reduced tumor mobility is strongly associated with worse prognosis.

### 3.10 Survival Analysis of Tumors With and Without a Pedicle

The presence of a tumor pedicle significantly affected patient survival (log‐rank *p* < 0.001) (Figure 4E). Patients with non‐pedunculated tumors faced a substantially higher mortality risk compared to those with pedunculated tumors (HR = 3.961, 95% CI: 1.925–8.152), indicating that pedunculated tumors are associated with improved prognosis.

### 3.11 Tumor Border Clarity and Survival Prognosis

Tumor border definition significantly influenced outcomes (log‐rank *p* < 0.001) (Figure 4F). Patients with well‐defined tumor borders had a markedly lower risk of death than those with poorly defined borders (HR = 0.086, 95% CI: 0.039–0.192). This finding suggests that clear tumor borders are an important indicator of favorable prognosis.

## 4 Discussion

This study retrospectively analyzed clinical data, echocardiographic features, pathological findings, and follow‐up information from 171 patients with right heart masses (2010–2023). We systematically examined the diagnostic characteristics, prognostic factors, and the utility of echocardiography in evaluating these right heart masses. The results revealed significant heterogeneity in the pathological types of right heart masses, with myxomas (63.4%) representing the predominant benign tumor type and angiosarcomas (63.3%) constituting the most common malignant tumor type. Survival analysis demonstrated that patients with benign tumors of the right heart had significantly better overall survival compared to those with malignant tumors, metastatic tumors, or thrombi (*p* < 0.001). Multivariate analysis identified tumor type (HR = 10.072), pedicle presence (HR = 0.383), and metastasis (HR = 3.210) as independent prognostic factors. Specifically, patients with primary malignant tumors of the right heart, absence of a pedicle, and metastasis exhibited significantly poorer outcomes. Echocardiography provided essential information for mass localization, characterization of morphology and mobility, and analysis of hemodynamic effects. Contrast‐enhanced imaging offered additional value in differentiating vascularized tumors from avascular thrombi. An exploratory analysis was performed in the subset of 19 patients who underwent contrast‐enhanced echocardiography. The enhancement characteristics showed potential utility in suggesting hypervascular malignant tumors versus hypovascular lesions; however, given the small sample size, this finding should be interpreted as preliminary and requires validation in larger, prospective studies. These findings underscore the importance of echocardiography as a primary, noninvasive tool for the initial evaluation and risk stratification of right heart masses. Its real‐time capabilities are complementary to the detailed anatomical and tissue characterization provided by CT or MRI, and an integrated multi‐modality imaging approach is often necessary for optimal management [7]. Within this process, echocardiography, as the first‐line tool, plays a role that extends beyond mere detection to include initial risk stratification. Our findings resonate with recent seminal work by Paolisso et al., solidifying the value of specific echocardiographic markers in discriminating malignancy. Paolisso et al. [8] developed a weighted echocardiographic score identifying six independent predictors of malignancy: infiltration, moderate‐to‐severe pericardial effusion, polylobate shape, sessile attachment, inhomogeneity, and non‐left localization. Our study further confirms that simply counting the number of these features (a threshold of ≥ 3) yields exceptionally high diagnostic accuracy, significantly enhancing the clinical utility of echocardiography. Furthermore, the Classification Tree Analysis (CTA) proposed by Paolisso et al. [9] offers a complementary, stepwise diagnostic algorithm that prioritizes infiltration as the primary discriminator. The convergence of evidence from this score‐based and CTA‐based approach provides a robust framework for sonographers. By systematically evaluating these markers, clinicians can efficiently risk‐stratify cardiac masses, thereby optimizing the subsequent diagnostic pathway: managing lesions with few suspicious features more confidently towards benign pathways or direct surgery, while promptly triaging high‐risk masses for advanced imaging. This refined approach ultimately aims to minimize diagnostic delays and ensure patients receive the most appropriate treatment in a timely manner.

Our findings reveal both similarities and differences compared to previous studies. Junaid et al. [10] reported that myxomas constitute 70%–80% of primary benign cardiac tumors; however, their proportion in our cohort was slightly lower, likely due to the lower incidence of right atrial myxomas, as left atrial myxomas are more common [11]. Notably, angiosarcomas accounted for 63.3% of malignant tumors in our study, a proportion significantly higher than the 30%–40% reported in broader literature [12]. This discrepancy may be explained by selection bias, as our study exclusively included right heart masses. Prior studies support the predominance of angiosarcomas among malignant right heart tumors [13]. Additionally, patients with malignant tumors had significantly lower survival rates than those with benign tumors (*p* < 0.001), consistent with Liu et al.'s [2] findings on the aggressive nature and high mortality of cardiac malignancies.

This study demonstrated that the diagnostic accuracy of contrast‐enhanced ultrasound for hypervascular masses was 87.5%, closely aligning with Xia et al.'s findings (82.4%) [14]. However, the diagnostic accuracy for hypovascular masses was 77.8%, slightly lower than the reported 100% [14]. This difference may be attributed to the small sample size (only 19 cases underwent contrast‐enhanced imaging) and the heterogeneous vascularity of certain benign tumors, such as fibromas. Importantly, the application of contrast‐enhanced ultrasound significantly improved diagnostic accuracy compared to conventional echocardiography [6]. Furthermore, this study verified that pedicle attachment, well‐defined borders, and high mobility are key indicative features of benign masses, consistent with the “ultrasound characteristics model for benign masses” proposed by Pasquale et al. [9].

Multivariate Cox regression analysis identified tumor type, pedicle presence, and metastasis as independent prognostic factors, while tumor borders and mobility were not statistically significant (*p* > 0.05). This contrasts with Yin et al.'s findings, which identified age as an independent predictor of malignancy risk [15]. The disparity may stem from methodological differences: our study adopted the United Nations World Health Organization's age classification rather than strict age stratification, potentially introducing bias. Metastasis had a significant impact on prognosis (HR = 3.210), consistent with Orlandi et al.'s findings on cardiac metastatic tumor survival (HR = 2.667) [16]. Similarly, Liu et al. [2] reported that malignant cardiac tumors are associated with poor survival outcomes, further supporting our conclusion that primary malignancies and metastases are key predictors of mortality.

One of the most clinically coherent findings of this study is the identification of the presence of a pedicle as an independent protective prognostic factor. This result is highly consistent with biological plausibility: a pedunculated morphology typically suggests non‐infiltrative growth with a well‐defined plane from the cardiac wall, which likely translates into higher rates of complete surgical resection and, consequently, improved patient outcomes. This underscores the importance of echocardiographic morphological characterization for risk stratification [9, 17]. Echocardiography offers unique diagnostic advantages through real‐time visualization of tumor dynamics. For highly mobile masses, multiplanar imaging is recommended to assess pedicle presence. These findings align with Rudski et al.'s [1] emphasis on echocardiography's value in evaluating right heart structures and detecting masses, particularly its diagnostic accuracy in characterizing tumor morphology and hemodynamic effects.

Among nine patients diagnosed with thrombi, three were initially misdiagnosed with tumors due to overlapping ultrasound features (e.g., echogenicity and mobility), consistent with Dhawan et al.'s observations [18, 19]. Contrast‐enhanced echocardiography successfully identified five avascular thrombi, highlighting its adjunct diagnostic value for thrombus detection, as advocated by Levine et al. [20] Compared to Gupta et al.'s [3] focus on malignant cardiac tumors, our study encompassed a broader spectrum of right heart masses, including benign tumors, thrombi, and non‐tumorous lesions. This inclusive approach enhances understanding of differential diagnosis and prognostic stratification. Additionally, our findings on echocardiography's ability to detect tricuspid valve obstruction and hemodynamic changes reinforce its clinical utility, as highlighted by O'Donnell et al. [5] Collectively, these insights advance the clinical understanding of right heart masses.

### 4.1 Limitations

Despite its strengths, this study has several limitations. First, the principal limitation of this study is selection bias inherent to the inclusion criterion of pathological confirmation. Our cohort therefore represents a pathologically confirmed, surgically biased case series. This may lead to an overrepresentation of lesions amenable to intervention (e.g., myxomas, primary sarcomas) and an underrepresentation of entities typically managed without pathological verification (e.g., thrombi diagnosed by clinical and imaging follow‐up) or advanced cases not undergoing biopsy. Consequently, the generalizability of our findings to all patients with right heart masses may be limited. Another key limitation of this study is the restriction of the cohort to only pathologically confirmed cases. While this criterion ensures diagnostic certainty, it introduces selection and verification bias. Our cohort is therefore skewed towards lesions accessible to surgery or biopsy. This may lead to an overrepresentation of tumors requiring intervention (e.g., myxomas, sarcomas) and an underrepresentation of entities often managed medically or followed clinically (e.g., typical thrombi, advanced malignancies not subjected to biopsy), thereby limiting the generalizability of our findings to the broader population of patients with right heart masses. Second, the study relied primarily on echocardiography as the imaging modality, which, while highly effective, may have limitations in detecting certain tumor characteristics compared to other advanced imaging techniques such as cardiac MRI or CT. Furthermore, as a retrospective study, we could not quantitatively assess the inter‐ and intra‐observer variability for the evaluated echocardiographic morphological characteristics (e.g., mobility, border definition, attachment). This methodological limitation may impact the consistency of measurements for more subjective parameters. Future prospective studies should incorporate standardized reproducibility assessments. Third, the lack of molecular or genetic analyses of the tumors limits our understanding of their underlying pathogenesis and potential therapeutic targets. For instance, genomic profiling could reveal actionable mutations or pathways that could inform targeted therapies. An additional limitation of this study is the lack of systematic assessment of the impact of right heart masses on right ventricular function. Acquiring and analyzing parameters of RV systolic and diastolic function (e.g., TAPSE, FAC, RV global longitudinal strain) and RV‐PA coupling indices (such as TAPSE/sPAP) would provide invaluable insights into whether the mass effect, obstruction, or myocardial infiltration leads to RV dysfunction or uncoupling. This information, bearing genuine pathophysiological and prognostic significance, represents a critical direction for future prospective studies. Fourth, the relatively small sample size for certain subgroups, such as primary malignant tumors, may limit the generalizability of our findings to broader populations. Finally, the study did not evaluate the impact of emerging treatment modalities, such as immunotherapy or targeted therapies, on patient outcomes.

## 5 Conclusion and Future Perspectives

This retrospective analysis of 171 patients with right heart masses identified tumor type, stalk presence, and metastatic status as key independent prognostic factors, while highlighting the critical role of echocardiography in characterizing morphological and hemodynamic properties. The study's limitations provide a framework for future investigations. First, prospective multicenter studies are needed to minimize bias and validate these findings through larger cohorts. Second, research should focus on integrating multi‐modal imaging, particularly three‐dimensional echocardiography, cardiac MRI, and CT, to develop more accurate diagnostic systems. Additionally, elucidating molecular mechanisms through genomic analyses is essential for understanding pathogenesis and identifying therapeutic targets. Concurrently, risk stratification models incorporating clinical, imaging, and pathological data would facilitate personalized treatment strategies. Finally, long‐term follow‐up studies evaluating emerging therapies, such as targeted treatments and immunotherapies, are warranted with emphasis on early detection and recurrence prevention in high‐risk patients. Advancements in these areas promise to significantly improve clinical management of right heart masses.

## Author Contributions

**Lei Liu:** investigation, data curation, formal analysis, writing – original draft. **Peng Teng:** investigation, data curation, formal analysis, writing – original draft. **Fudong Fan:** investigation, data curation, formal analysis, writing – original draft. **Hui Chen:** investigation, resources, writing – review and editing. **Yanfei Mo:** formal analysis, software, validation, writing – review and editing. **Jing Yao:** validation, visualization, supervision, writing – review and editing. **Xuelin Yang:** conceptualization, supervision, project administration, writing – review and editing, funding acquisition. **Aijuan Fang:** conceptualization, supervision, project administration, writing – review and editing.

## Conflicts of Interest

The authors declare no conflicts of interest.

## Data Availability

The datasets used and/or analyzed during the current study are available from the corresponding author (Aijuan Fang, fajglyy0607@126.com) on reasonable request.
