# Correction to “Clinical and Echocardiographic Insights Into Right Heart Masses: A Retrospective Study in China (2010–2023)”

**DOI:** 10.1002/cam4.71716

**Published:** 2026-03-10

L. Liu, P. Teng, F. Fan, et al. “Clinical and Echocardiographic Insights Into Right Heart Masses: A Retrospective Study in China (2010–2023),” *Cancer Medicine* 15, no. 1 (2026): e71530, https://doi.org/10.1002/cam4.71530.

All the author units involved in Nanjing Drum Tower Hospital:

“Drum Tower Hospital Affiliated to Nanjing University Medical School, Nanjing, China.”

Due to the problem of translation mode, it needs to be changed to:

“Nanjing Drum Tower Hospital, Affiliated Hospital of Medical School, Nanjing University, Nanjing, P.R. China”

We apologize for this error.

